# Efficacy and side effects of long-term treatment strategies of canine primary hypoadrenocorticism

**DOI:** 10.3389/fvets.2025.1735233

**Published:** 2026-01-05

**Authors:** Deborah Anna Dobosz, Yury Zablotski, Astrid Wehner

**Affiliations:** Clinic of Small Animal Medicine, Centre for Clinical Veterinary Medicine, Ludwig Maximilian University of Munich, Munich, Germany

**Keywords:** desoxycorticosteronepivalate, desoxycortonpivalate, DOCP, dogs, fludrocortisone, hypoadrenocorticism, prednisolone, questionnaire

## Abstract

**Background:**

Hypoadrenocorticism (HA) with hyponatremia and hyperkalemia represents primary disease and indicates cortisol and aldosterone insufficiency. Treatment involves replacement of mineralocorticoids and glucocorticoids. There are currently no studies comparing the efficacy and side effects of different treatment protocols.

**Objectives:**

The aim of this study was to compare different treatment protocols for dogs with hyponatremic and hyperkalemic HA regarding their efficacy and side effects.

**Animals:**

Two hundred forty-four dogs with HA with electrolyte anomalies.

**Methods:**

Data were collected via a standardized online questionnaire (available in english and german), which caregivers of affected dogs completed. The questionnaire was given to clients and was spread in online media.

**Results:**

A total of 244 complete questionnaires were received (109 german and 135 english). Of those 244 participating dogs, 126 were female and 118 were male. Based on the treatment applied, the following groups were assigned: prednisolone and desoxycorticosterone pivalate (DOCP, Zycortal®) (ZP, *n* = 167), prednisolone and desoxycorticosterone pivalate (DOCP, Percorten-V®) (PP, *n* = 39), prednisolone and fludrocortisone (FP, *n* = 23) and fludrocortisone (F, *n* = 15). The median prednisolone dose was 0.07 mg/kg/day in the ZP and 0.08 mg/kg/day in the FP group and was slightly lower in the PP group with 0.04 mg/kg/day. Median DOCP dose was 0.88 mg/kg every 28 days in the ZP group and 0.78 mg/kg every 28 days in the PP group. Fludrocortisone was dosed with 0.02 mg/kg/day in the FP and F group. All treatment strategies led to a normal activity, quality of life of dog and caregiver, and achieved satisfaction with therapy.

**Conclusion:**

Good clinical disease control can be achieved with any of the above strategies. Applied DOCP dose were lower than previously reported and can be combined safely with low prednisolone dosages.

## 1 Introduction

Hypoadrenocorticism (HA) is considered a rare endocrinopathy in the dog (1, 2). It can be caused by failure of the adrenal cortex (primary disease) or by failure of the pituitary gland and consecutive atrophy of the adrenal cortex (secondary disease). Primary disease is most common and is thought to be mainly caused by immune-mediated destruction of the adrenal cortex (3). Cortisol deficiency will result from a loss of function of the zona fasciculata and zona reticularis. Destruction of the zona glomerulosa will result in aldosterone deficiency (4). Almost all dogs with primary HA will have glucocorticoid deficiency and most dogs will have mineralocorticoid deficiency as well (5).

A genetic predisposition to HA is suspected in dogs which is comparable to the situation in humans. HA is most common in young to middle-aged female dogs. Several breeds have been reported to be at an increased risk, including Standard Poodle, West Highland White Terrier, Soft Coated Wheaten Terrier, Rottweiler, Great Dane, Portuguese Water Dog, Bearded Collie and Nova Scotia Duck Tolling Retriever (4, 6, 7).

Glucocorticoid deficiency leads to nonspecific symptoms such as weakness, anorexia, vomiting, weight loss and diarrhea, which all can occur episodically (8, 9). Aldosterone deficiency is the cause of potentially life-threatening biochemical abnormalities such as hyperkalemia, hyponatremia and metabolic acidosis (10, 11). The current gold standard for the diagnosis of HA is the ACTH stimulation test, which measures serum cortisol concentrations immediately before and one hour after intravenous or intramuscular administration of synthetic ACTH (4, 8, 12–14).

Treatment of HA almost always requires lifelong replacement of glucocorticoids. In dogs, mainly prednisolone is given. In case of aldosterone deficiency, replacement with synthetic mineralocorticoids is indicated. Desoxycortone pivalate or desoxycorticosterone pivalate (DOCP) are injectable depot formulations whereas Fludrocortisone is available in tablets and is approved for humans. Dogs can develop iatrogenic hypercortisolism if treated with prednisolone and/or fludrocortisone. Persistent hypocortisolism during treatment is rare. Failure to normalize sodium and potassium can occur with Fludrocortisone treatment. Many studies have demonstrated that DOCP is associated with less side effects, better electrolyte control, and a better caregiver satisfaction compared to fludrocortisone acetate (15–19). Currently, there are two DOCP formulations approved for dogs, Percorten-V®, which is injected intramuscular and Zycortal®, which is injected subcutaneously (4, 18, 20–22, 40).

Assessment of quality of life (QoL) plays a central role in the management of chronic diseases. In veterinary medicine, caregivers are pivotal in this assessment. QoL has already been examined in endocrinopathies such as Cushing’s syndrome (23), diabetes mellitus (24), and HA (25).

There are currently no data comparing the effectiveness of the different formulations to replace aldosterone. Therefore, this standardized web-based questionnaire was designed to evaluate if clinical symptoms in dogs resolve or if some symptoms persist during treatment of HA taking the treatment protocol into account. General data of the dog, and other health related issues before and during treatment were also assessed.

## 2 Materials and methods

### 2.1 Ethics and data protection

This research received ethical approval from the Ethics Committee of the Faculty of Veterinary Medicine, LMU Munich, Germany (reference number 292–18-10-2021). At the beginning of the questionnaire, dog caregivers gave their consent to participate in the study. Participants could remain anonymous if they wished to do so. Any contact information voluntarily provided by dog caregivers was removed prior to data cleaning. Statistical evaluation was performed with anonymized data.

### 2.2 Data collection

The study was designed as a prospective survey. A standardized web-based questionnaire was developed, which was accessible online in English and German. To ensure clarity and comprehension, the preliminary version was tested by 10 dog caregivers whose feedback was incorporated into the final version. Participation was limited to caregivers of dogs with a confirmed diagnosis of HA with electrolyte abnormalities (formerly addressed as ‘typical’) established by a veterinarian based on ACTH stimulation testing. Furthermore, only dogs that had been undergoing continuous treatment for at least three months prior to questionnaire completion were eligible. Each questionnaire was to be completed only once per eligible dog. All participating caregivers confirmed that they were of legal age.

Caregivers of eligible dogs were invited to participate in the study. An introductory letter containing the link to the study website was available on the homepage of the Clinic of Small Animal Medicine (Ludwig Maximilian University, Munich) and was distributed via social media platform and moreover sent via e-mail to endocrinologists of universities in different countries in Europe. The data from the online questionnaire was collected using the evasys platform (version 9.1, 2,464). Data were exported as comma-separated values (CSV) files and imported into Microsoft Excel® for Mac, version 16.96.1 (Microsoft Corporation, Redmond, WA, USA) for further processing and initial data cleaning. The German-language questionnaire was released on September 5, 2021, and the English-language questionnaire on December 23, 2022. Both questionnaires were available until October 30, 2023.

The questionnaire was divided into different sections. The first section provided general information about the study and outlines the inclusion criteria. The second section was designed to collect information about the caregiver regarding their place of residence, their age and gender. These information were optional. The third section seeked general questions about the dog, including sex, breed, age, origin, ectoparasite prophylaxis, deworming, vaccinations, and weight.

The fourth section focused on questions relating to the diagnosis and treatment of HA. The fifth section covered general clinical signs such as drinking behavior, urine volume, appetite, stool consistency, defecation frequency panting, skin and coat condition. The sixth section dealt specifically with possible GI signs and their treatment before HA treatment was initiated, while the seventh section covered the same aspects during HA treatment. The eighth section focused on comorbidities and their management, while the final section explored the dog’s health, activity, general behavior and well-being of dog and caregiver.

Multiple Choice questions (‘Yes’ or ‘No’ or ‘Unknown’) were used for the assessment of performed diagnostics, treatment strategies for HA, comorbidities, and their management. Extended Multiple-Choice questions were used for ectoparasite prophylaxis, deworming and vaccination (‘Yes’, ‘No’‚ ‘No, but,… ‘).

Signs which potentially could improve or worsen were rated on a 7-point Likert-type scale, which ranged from ‘severely increased’ through ‘normal’ to ‘severely decreased’. This applied for signs such as satisfaction with treatment, appetite, development of weight. For signs that could range from normal to abnormal, such as drinking behavior, urination frequency, or skin and hair condition, a 4-point scale was used. Response options included ‘severely increased’, ‘moderately increased’, ‘slightly increased’, and ‘normal’. Likewise, a meaningful answer to questions on health and well-being could only be ‘normal’ or ‘decreased’, which were also assessed on a 4-point scale.

Stool consistency was rated according to the Chronic Enteropathy Clinical Activity Index (CCECAI) (26). Frequency of GI problems was also recorded (more than 3 times per week or less).

For data analysis, the original rating scales were simplified. The 7-point scales were collapsed into three categories (‘decreased’, ‘normal’, ‘increased’), and 4-point scales into two categories (‘normal’, ‘increased’). If respondents were unsure about a question, the option ‘unknown’ was available and retained as a separate category in the dataset. Additionally, respondents had the opportunity to provide comments in a free text field. These entries were reviewed, summarized, and included in the analysis where applicable (e.g., for specifying the dose and/or interval of a medication).

Treatment duration in days was determined based on the reported date of diagnosis and the completion date of the questionnaire; dogs that had already died or were euthanized were excluded from this calculation.

### 2.3 Statistical analysis

Statistical analyses were conducted using GraphPad Prism for Windows (version 5.04, GraphPad Software, San Diego, CA, USA) and R (version 4.5.0 (2025-04-11), R Foundation for Statistical Computing, Vienna, Austria).

Descriptive statistics and frequency distributions were calculated using Microsoft Excel® for Mac, version 16.96.1 (Microsoft Corporation, Redmond, WA, USA). For data cleaning, the dosing intervals of prednisolone and fludrocortisone therapy (e.g., every 8, 12, or 24 h) was reviewed and categorized based on the information provided by the caregivers. If concomitant treatments were provided (such as probiotics, antibiotics, cobalamin supplementation, gastric protectants, and other medications), this information was systematically categorized before statistical analysis.

Spearman rank correlation analyses was performed using GraphPad Prism to assess associations between treatment duration and prednisolone dosage (mg/kg/day) in all prednisolone-treated groups (ZP, PP, FP), between treatment duration and DOCP dosage (mg/kg) in all DOCP-treated groups (ZP, PP), and between treatment duration and fludrocortisone dosage (mg/kg/day) in fludrocortisone-treated groups (FP, F). Further analyses examined correlations between body weight (kg) and the respective medication dosages in each treatment group, including prednisolone dosage in prednisolone-treated dogs (ZP, PP, FP), DOCP dosage in DOCP-treated dogs (ZP, PP), and fludrocortisone dosage in dogs treated with fludrocortisone (FP, F). To evaluate the relationship between treatment duration and the presence of polyuria (PU) or polydipsia (PD), Wilcoxon matched-pairs signed rank tests were performed within each treatment group (ZP, PP, FP, F), along with Spearman rank correlation analyses.

To assess intra-individual changes in clinical signs (GI signs, PD, PU) before and since HA treatment, the Wilcoxon matched-pairs signed rank test was used for paired, non-normally distributed data. These tests were performed within each treatment group (ZP, PP, FP, F).

To evaluate differences in the occurrence of clinical signs, satisfaction and reduced QoL (dog and caregivers) between treatment groups, a logistic regression model (generalized linear model with logit link) was applied using R. Estimated marginal probabilities were calculated, visualized as frequencies, and pairwise group contrasts were performed to obtain odds ratios and 95% confidence intervals, using the emmeans package with Tukey adjustment for multiple comparisons. Statistical significance was defined as *p* < 0.05. Intervals were back-transformed from the log-odds scale.

## 3 Results

### 3.1 Animals

In total, 393 questionnaires were received. Questionnaires were excluded if their results were not consistent with HA, if the duration of treatment was less than 3 months, if HA without electrolyte disturbances (hyponatremia and hyperkalemia) or iatrogenic HA was present, or if other glucocorticoids than prednisolone were used.

244 questionnaires on dogs with electrolyte anomalies (hyponatremic, hyperkalemic) HA were included in the analysis. Based on the treatment provided, the following groups were created: Zycortal® and prednisolone (ZP, *n* = 167), Percorten-V® and prednisolone (PP, *n* = 39), fludrocortisone and prednisolone (FP, *n* = 23), and fludrocortisone alone (F, *n* = 15). One hundred nine (45%) participants answered the german questionnaire, and 135 (55%) participants answered the english questionnaire. Table 1 gives an overview of the demographic distribution of dogs.

**Table 1:** Demographic distribution of owners filling in the questionnaire.

Country	Questionnaires received
Germany	98
USA	81
UK	25
Canada	15
Australia	5
Austria	4
Switzerland	3
Norway	1
New Zealand	1
France	1
Romania	1
Argentina	1
Netherlands	1
Ireland	1
Israel	1
Italy	1
Unknown (German questionnaire)	4
Total	244

Out of the 244 dogs, 126 were female. Of these, 20 were intact, while 106 were spayed. Among the 118 male dogs, 18 were intact, and 100 were neutered. The median age of the dogs at the time the questionnaire was completed was 7.0 years (IQR: 4.25–10.0 years; range 0–18 years). At the time of diagnosis, the median age was 3 years (IQR: 2–5 years; range 0–15 years). The median treatment duration across all groups was 786 days (IQR 335–1,570 days). When analyzed separately, the median treatment duration was 761 days (IQR 373–1,542 days) in the ZP group, 1,467 days (IQR 264–2,575 days) in the PP group, 688 days (IQR 204–1,178 days) in the FP group, and 488 days (IQR 238–785 days) in the F group.

The median weight of the dogs at the time of completing the questionnaire was 23 kg (IQR 17–30 kg; range 283 kg).

One hundred and four (43%) dogs were mixed breeds, 24 (10%) were Poodles, 15 (6%) were Poodle cross-breed (e.g., Doodles, Poos), 12 (5%) were Labrador Retrievers, and 11 (5%) were Bearded Collies. Four dogs (2%) each belonged to the breeds Border Collie, Cocker Spaniel, Bernese Mountain Dog, and Bolonka Zwetna, and 3 dogs were German pinschers. The remaining 59 dogs comprised 46 other breeds. A detailed overview of the breeds is provided in Table 2.

**Table 2:** Overview of dog breeds.

Breed	Number of dogs
Cross-breed /mixed breed	104
Poodles (11 kg - 40 kg)	24
Doodles/poos	15
Labrador retriever	12
Bearded collie	11
Border collie	4
Cocker spaniel	4
Bernese mountain dog	4
Bolonka zwetna	4
German pinscher	3
Other breeds	59

Nineteen out of 244 dogs (8%) had been euthanized or had passed away at the time the questionnaire was completed. These dogs had lived a median of 13 years (IQR 10.5–14.5). In the ZP group, six dogs had been euthanized or had died. One dog had died from a neoplastic disease at the age of 12, two dogs at the age of 13 and 14 due to geriatric causes. One dog at the age of 9 died from unknown causes. One dog died due to HA at the age of 12. One dog was euthanized due to systemic immune-mediated disease at the age of 8. In the PP group, three dogs had been euthanized or had died. One dog with unknown age had been euthanized due to a neoplastic disease, another had died at the age of 14 due to geriatric causes, and the third dog had died due to urinary tract obstruction at the age of 9. In the FP group, eight dogs had been euthanized or had died. Among these, two dogs had been euthanized due to neoplastic diseases at the ages of unknown and 14 years. One 15-year-old dog had died from hepatic disease, and another dog at the age of 13 due to geriatric causes. One dog, aged 16, had died of unknown causes. One dog had died at the age of 8 years due to comorbidities, while two dogs aged 14 and 15 years had no information available regarding the cause of death. In the F group, two dogs had been euthanized or had died. One dog had died at the age of 18 due to renal disease, and the other succumbed to a neoplastic disease at 13 years of age.

### 3.2 Treatment

#### 3.2.1 Glucocorticoid dosage

A total of 229 dogs belonging to the groups ZP, PP, FP were treated with prednisolone. Information concerning the dose was available for 223 dogs. The median prednisolone dose was 0.06 mg/kg/day. Dogs in the ZP group received a median prednisolone dose of 0.07 mg/kg/day. Dogs in the FP group received a median dose of 0.08 mg/kg/day, and dogs in the PP group received significantly less prednisolone (0.04 mg/kg/day, *p* < 0.0001) compared to the FP and ZP group. The doses are listed in Table 3.

**Table 3:** Prednisolone dosages in the ZP, PP, and FP group.

Prednisolone dosage	ZP *n*^1^ = 163	PP *n* = 37	FP *n* = 22	Comparison
Median (IQR^2^) mg/kg/d^3^	0.07 (0.04–0.09)	0.04 (0.03–0.06)	0.08 (0.05–0.15)	FP vs^4^. PP: OR^5^: 1.92 *p*-value^6^: 0.0012* FP vs. ZP: OR: 1.25 *p*-value: 0.322 PP vs. ZP: OR: 0.65 *p*-value: 0.0015*
Mean (Range) mg/kg/d	0.08 (0.01–0.1)	0.05 (0.01–0.1)	0.13 (0.04–0.59)	

^1^n, number; ^2^IQR, interquartile range; ^3^d, day; ^4^vs, versus; ^5^OR, odds ratio; ^6^*p*, level of significance; *significant *p*-value.

Prednisolone dosages (mg/kg/day) in the ZP, PP, and FP groups. Data are presented as median (interquartile range, IQR) and mean (range). Comparisons between groups were performed using logistic regression models, with results given as odds ratios and corresponding *p*-values.

Of the 229 dogs, 209 received prednisolone once daily, 16 dogs received it twice daily, and one dog received it every 8 h. For three dogs, no information regarding the dosing frequency was provided. A statistically significant negative correlation was also found between treatment duration and prednisolone dosage (rs = −0.147, *p* = 0.005). A statistically significant negative correlation was observed between prednisolone dosage (mg/kg/day) and body weight (kg) (rs = −0.272, *p* = 0.00005).

#### 3.2.2 Mineralocorticoid dosages

The median Zycortal® dose was 0.88 mg/kg (IQR: 0.52–1.26 mg/kg), and the median Percorten-V® dose was 0.78 mg/kg (IQR: 0.57–1.27 mg/kg). The median Zycortal® dosing interval was 28 days (IQR 28–30 days; range 21–59.5 days), and the median Percorten-V® interval was also 28 days (IQR 28–29 days; range 25–30 days). There was no statistically significant difference in the administered doses or dosing intervals between ZP and PP dogs.

A total of 38 dogs received fludrocortisone, with 23 dogs allocated to the FP group and 15 to the F group. The average fludrocortisone dosage was 0.02 mg/kg per day. There was no statistically significant difference in fludrocortisone dosage between the FP and F groups.

In the FP group, 16 dogs received fludrocortisone every 12 h, 6 dogs once daily, and for 1 dog the dosing interval was not reported. In the F group, 14 dogs received fludrocortisone every 12 h and 1 dog once daily. There was no statistically significant difference in the fludrocortisone dosages between the FP and F groups.

A statistically significant negative correlation was observed between treatment duration and DOCP (Zycortal® and Percorten-V®) dosage (rs = −0.359, *p* < 0.0001). No significant correlation was found between treatment duration and fludrocortisone dosage (rs = 0.056, *p* = 0.795). No significant correlation was found between DOCP (Zycortal® and Percorten-V®) dosage and body weight (rs = −0.099, *p* = 0.200), nor between fludrocortisone dosage and body weight (rs = −0.033, *p* = 0.794).

Tables 4, 5 provide a more detailed breakdown of the DOCP and fludrocortisone dosages.

**Table 4:** DOCP dosage.

DOCP^1^ dosage	ZP *n*^2^ = 138	PP *n* = 37	Comparison
Median (IQR^3^) mg/kg/d^4^	0.88 (0.51–1.26)	0.78 (0.57-–1.27)	ZP vs^5^. PP: *p*-value^6^: 0.64
Mean (Range) mg/kg/d	0.96 (0.22–3.0)	0.9 (0.35–1.86)	

^1^DOCP, desoxycorticosterone pivalate; ^2^n, number; ^3^IQR, interquartile range; ^4^d, day, ^5^vs, versus; ^6^*p*, level of significance.

DOCP dosages (mg/kg/day) in the ZP and PP groups. Values are presented as median (interquartile range, IQR) and mean (range). Comparisons between groups were performed using appropriate statistical tests, and results are displayed with corresponding *p*-values.

**Table 5:** Fludrocortisone dosage.

Fludrocortisone dosage	FP *n*^1^ = 21	F *n* = 15	Comparison
Median (IQR^2^) mg/kg/d^3^	0.02 (0.01–0.03)	0.02 (0.02–0.02)	FP vs^4^. F: *p*-value^5^: 0.89
Mean (Range) mg/kg/d	0.025 (0.007–0.07)	0.021 (0.01–0.04)	

^1^*n*, number; ^2^IQR, interquartile range; ^3^d, day; ^4^vs, versus; ^5^*p*, level of significance.

Fludrocortisone dosages (mg/kg/day) in the FP and F groups. Values are presented as median (interquartile range, IQR) and mean (range). Comparisons between groups were performed using appropriate statistical tests, and results are displayed with corresponding p-values.

### 3.3 Assessment of disease control: activity, quality of life (QoL), and satisfaction

The activity level was predominantly rated as normal within each group since therapy. According to their caregivers, dogs in the ZP, FP and F group had the highest occurrence of reduced activity with 13.94% (*p* < 0.0001), 13.63% (*p* = 0.0018), and 13.33% (*p* = 0.0024), respectively. The lowest rate was present in the PP group with 2.56% (*p* < 0.0001). However, no statistically significant differences were found in pairwise comparisons between groups (all *p* > 0.05).

QoL of dogs was mainly rated as normal in all groups. Since therapy, 26.1% of dogs in the FP group were reported with reduced QoL (*p* = 0.0186), followed by 10.78% in ZP (*p* < 0.0001), 6.67% in F (*p* = 0.001), and 2.56% in PP (*p* < 0.0001). None of the pairwise group comparisons reached statistical significance (all *p* > 0.05).

More caregivers reported to have a reduced QoL since therapy. Reduced QoL was reported by 33.3% of caregivers in the FP group (*p* = 0.305), 23.17% in ZP (*p* < 0.0001), 12.5% in F (*p* = 0.028), and 11.11% in PP (*p* = 0.0002). Despite these differences, none of the pairwise comparisons between groups reached statistical significance (all *p* > 0.05).

All groups demonstrated high satisfaction levels with the treatment outcome. A lack of satisfaction was reported by none of the caregivers in the FP and F group (*p* = 0.0069 and *p* = 0.0004, respectively), 2.56% in the PP (*p* = 0.0001), and 3.9% in the ZP group (*p* < 0.0001). No statistically significant differences were observed between groups in pairwise comparisons (all *p* > 0.05).

A detailed summary of all findings related to reduced activity, quality of life, and satisfaction are provided in the supplemental data (Supplementary Table S1).

### 3.4 GI signs before and since treatment

Vomiting, altered fecal consistency, reduced appetite, and weight loss were assessed as a composite variable and reported both before and since therapy across all treatment groups (ZP, PP, FP, F).

In the ZP group, significantly more dogs were affected by GI signs before than during treatment (before: 137/164 (83.5%), since: 61/164 (37.2%); *p* < 0.0001). A similar pattern was seen in the PP group (before: 34/39, 87.2%; since: 9/39, 23.1%; *p* < 0.0001), in the FP group (before: 20/21, 95.2%; since: 10/23, 43.5%; *p* = 0.0011), and in the F group (before: 14/15, 93.3%; since: 4/15, 26.7%; *p* = 0.0019).

A logistic regression model was applied to assess differences between groups in the likelihood of GI signs since treatment. The estimated proportions were 42.7% in the FP group (*p* = 0.4750), 37.1% in ZP (*p* = 0.0011), 28.2% in F (*p* = 0.0651), and 23.3% in PP (*p* = 0.0013). *Post hoc* comparisons between groups revealed no statistically significant differences in odds ratios OR (all *p* > 0.05).

Supportive treatments such as probiotics, antibiotics, cobalamin supplementation, gastric protection and other medications were recorded across all groups before and since HA treatment. Probiotics and antibiotics were the most frequently administered supportive treatments, both before and since HA-therapy, whereas drugs for gastric protection were rarely used. Detailed descriptions are presented in the supplemental data (Supplementary Table S2).

Before HA therapy, a dietary change was documented in 52 dogs. Of these, 13 dogs (25.0%) showed an improvement in GI signs, 7 dogs (13.5%) worsening signs, and 32 dogs (61.5%) remained unchanged. Since HA therapy, a dietary change was reported in 125 dogs. Of these, 99 dogs (79.2%) showed an improvement, 11 dogs (8.8%) worsening signs, and 15 dogs (12.0%) remained unchanged.

### 3.5 Evolution of selected clinical signs during treatment

In this section, questions were assessed before and during treatment that could be related to HA itself or to a possible glucocorticoid excess such as PD, PU, and polyphagia.

When comparing the occurrence of PD and PU before and during therapy within each group, no statistically significant changes were detected (*p* > 0.05 for all). During treatment, a statistically significant between-group effect for PU was observed in the PP group (*p* = 0.034), with dogs being less frequently affected. Comparisons between groups revealed no significant differences for PD since therapy. In contrast, for polyphagia, significant within-group differences were observed between before and during treatment in all groups (ZP: *p* < 0.0001; PP: *p* = 0.0002; FP: *p* = 0.0006; F: *p* = 0.011), indicating that polyphagia was reported more frequently during therapy compared to before therapy. A significant between-group difference was also detected between the PP and ZP groups (*p* = 0.032) since therapy, with dogs in the PP group being less frequently affected.

An overview of the frequency of PD, PU, and polyphagia since therapy across all groups is provided in (Supplementary Table S3).

## 4 Discussion

This questionnaire aimed to evaluate the efficacy of different therapeutic strategies for the treatment of hyponatremic and hyperkalemic HA, by collecting data on the administered products, their dosages and treatment intervals as well to assess the efficacy and possible side effects of the provided treatment.

Canine HA is considered a rare endocrine disorder in dogs (1, 2, 27). Several therapeutic strategies are available for dogs with hyponatremic and/or hyperkalemic HA. Typically, a combination of a mineralocorticoid and a glucocorticoid medication is used (17, 28–31). In the case of fludrocortisone, monotherapy is also possible due to its confirmed glucocorticoid activity in dogs. Based on these therapeutic options, four treatment groups (ZP, PP, FP, F) were defined, and the information listed above were collected.

Mineralocorticoid replacement in dogs is commonly achieved with either fludrocortisone acetate or DOCP (32). The only approved veterinary product for mineralocorticoid deficiency is DOCP. The two commercially available DOCP formulations are Percorten-V® (Elanco, Indianapolis, IN, USA) and Zycortal® (Dechra Veterinary Products Nederland B. V., Handelsweg 25, 5,531 AE Bladel, The Netherlands) (21). Both products contain the same active ingredient but differ in their preservatives and surfactants (19). The route of administration also differs – Percorten-V® is given intramuscularly, while Zycortal® is administered subcutaneously (4, 18, 20–22). Due to these differences, two separate DOCP groups were defined in this study. According to the product labels, the recommended dose for both Percorten-V® and Zycortal® is 2.2 mg/kg every 25 days (15, 17), although a 4-week interval is also effective (33). Injection intervals of up to 94 and 99 days have also been reported (18, 34). Several studies have shown that a starting DOCP dose of 1.5 mg/kg is usually sufficient and can often be reduced over the course of treatment (15, 19, 33, 35). Another study suggested an initial DOCP dose of 1.1 mg/kg, though stating that this may not be adequate for all dogs (36).

In the present study, the median Zycortal® dose was 0.88 mg/kg and the median Percorten-V® dose was 0.78 mg/kg. No statistically significant difference was found between the two groups. These doses are considerably lower than the labeled dose (2.2 mg/kg) and also fall well below the commonly recommended starting dose of 1.5 mg/kg. It should be noted that the questionnaire asked about the current DOCP dose, and initial treatment doses may have been higher. This assumption is supported by a significant negative correlation between treatment duration and DOCP dose, indicating that the dosages tended to decrease as treatment continues. There was no correlation between dosages and body weight. The median injection interval for both Zycortal® and Percorten-V® was 28 days, which aligns with previous studies (33). Some authors recommend a fixed 28–31 day-schedule to improve caregiver compliance (32, 35). In the ZP group, the longest treatment interval reached approximately 8 weeks, which supports the documented long duration of action of Zycortal®.

In human medicine, oral fludrocortisone acetate is the most commonly used mineralocorticoid for patients with primary HA (17). This treatment has also been used in dogs for many years (17). Fludrocortisone has a half-life of approximately 11 h in dogs (37) and is administered orally once or twice daily (16). It possesses strong mineralocorticoid activity and moderate glucocorticoid activity in dogs (32), making additional glucocorticoid supplementation unnecessary and can be used as monotherapy in dogs with primary HA. The recommended starting dose of fludrocortisone is 0.01 mg/kg orally twice daily (17). In this study, the median fludrocortisone dose in both the FP and F groups was 0.02 mg/kg/day, consistent with standard dosing recommendations. It was typically administered twice daily. There was no association between dosage and body weight or treatment duration.

Prednisolone is considered the ideal glucocorticoid for maintenance therapy (38, 39). It is given once the diagnosis is established. Replacement is usually achieved with a physiologic dose of prednisone or prednisolone administered once daily (14, 40, 41). The typical maintenance dose ranges between 0.1 and 0.25 mg/kg/day (42, 43), although some larger breeds respond well to lower doses (e.g., ~0.05 mg/kg/day) (43). The optimal prednisolone dose should be tailored based on clinical signs. Lower doses are preferable to avoid side effects such as polyphagia, weight gain, PU/PD, and other glucocorticoid-related complications. If lethargy, anorexia, vomiting, or diarrhea reappear, the dose may need to be increase (8, 44). Temporary dose increases are also recommended in situations of physical or emotional stress, such as illness, surgery, travel, boarding, or exercise (1, 4, 39, 44–47). The dose is adjusted gradually based on clinical response and side effects (43). The goal is to titrate to the lowest effective dose that controls clinical signs while avoiding glucocorticoid deficiency (8).

Only questionnaires involving prednisolone as the glucocorticoid component were included in this study. Dogs in the ZP group received median 0.07 mg/kg/day, and those in the FP group received 0.08 mg/kg/day. Both doses were significantly higher than the PP group, where a median dose of 0.04 mg/kg/day was given. For long-term therapy of HA, prednisone dosages ranging from <0.05 to 0.4 mg/kg/day have been described (17). Studies, where prednisone was combined with DOCP, median doses between 0.10–0.35 mg/kg/day were given (33, 36). Studies, where prednisolone was combined with fludrocortisone, median doses between 0.06–0.5 mg/kg/day are documented (1, 8, 38, 44, 47, 48–51). The observation that the median prednisolone dosages in all three groups were within the lower ranges described in the literature highlights that very low glucocorticoid supplementation can be sufficient for long-term control. Nevertheless, it should be emphasized that our data refer to prednisolone, whereas most previous reports are based on prednisone, which needs to be considered when interpreting these comparisons. Prednisolone represents the pharmacologically active metabolite of prednisone, generated through hepatic conversion by the enzyme 11-*β*-hydroxysteroid dehydrogenase. For this reason, prednisolone is often preferred over its prodrug in dogs, as no additional hepatic biotransformation is required (52, 53). Although prednisone is rapidly converted to prednisolone in dogs, pharmacokinetic studies indicate that systemic exposure does not increase proportionally with higher prednisone doses (54). This study, representing the first systematic evaluation of prednisolone in combination with DOCP, demonstrated that prednisolone provides a predictable glucocorticoid effect, particularly in dogs with long-term treatment. To our knowledge, this study is among the first to report low median prednisolone doses in combination with DOCP, indicating that lower glucocorticoid supplementation may be sufficient for adequate long-term control in many dogs. It is noteworthy that in the ZP and FP groups, the maximum reported dose reached the anti-inflammatory range at 0.5 mg/kg/day, which was not observed in the PP group. A significant negative association was found between treatment duration and administered prednisolone dose (mg/kg/day), suggesting that prednisolone dosing tends to decrease over the course of treatment. Furthermore, it was shown in this study, that larger dogs received significantly lower doses in mg/kg/day.

Efficacy was assessed based on control of clinical signs and the occurrence of side effects. The clinical presentation of HA is often described as mild, nonspecific, and variable. The most common clinical signs include lethargy, reduced appetite, weight loss, vomiting, diarrhea, as well as PU and PD (4, 10, 12, 55, 56). For this reason, the questionnaire specifically collected information on activity level, gastrointestinal symptoms, and the presence of PU/PD. Currently, prednisolone dose adjustments are based solely on clinical signs. The most frequent consequence of prednisolone therapy is overtreatment, which can lead to iatrogenic Cushing’s syndrome (39). In one study, the most commonly reported side effects included PU/PD (26%), polyphagia (17%), coat changes (11%), excessive panting (9%), and weight gain (8%) (25). Based on these findings, the questionnaire also inquired about typical steroid-related side effects such as PU, PD, and polyphagia (57). Although DOCP is generally well tolerated, PU/PD has also been reported in some cases, usually due to concurrent glucocorticoid administration (17). Dogs in the PP group received the lowest median prednisolone dose, yet there were no indications of clinically relevant undertreatment. Neither reduced activity nor diminished QoL in either the dogs or their caregivers, nor increased dissatisfaction with treatment were more frequently reported in this group or any of the groups. This group achieved the highest treatment satisfaction and similar caregiver-reported QoL scores compared to the other groups, suggesting that low individualized glucocorticoid dosages were sufficient for clinical control. As lethargy is considered the most common clinical sign of untreated HA (55), this parameter was given special attention. During treatment, none of the groups (ZP, PP, FP, F) exhibited reduced activity, and no significant differences were found between groups.

QoL is an increasingly important metric in human medicine but remains poorly standardized in veterinary medicine. However, caregiver-perceived QoL is a major factor in treatment decisions for their pets, and a perceived reduction in QoL is often cited as a reason for euthanasia (58). Treatment changes or discontinuation may be unavoidable when caregivers perceive their own or their pet’s QoL as severely impaired – regardless of clinical success. Assessing QoL in animals remains challenging due to the lack of a universally accepted definition in veterinary medicine. Additional influencing factors such as age, breed, temperament, and individual variation must also be taken into account (59). Veterinarians play an essential role in QoL assessments in helping to optimize animal welfare (60). In this study, QoL was assessed using a non-validated tool, and no definition of QoL was provided. Furthermore, no veterinary clinical evaluation was included, which makes interpretation more difficult. However, currently, no HA-specific QoL tool exist (59). Our results are consistent with previous studies and show that most caregivers did not report a reduction in their own QoL or in that of their dog (25). There were no significant differences between treatment groups in these parameters. However, there was a trend toward a difference in dog QoL scores between the PP and FP groups. This trend should be further explored, as it may indicate suboptimal treatment control in the FP group. In another study, caregivers QoL was also rarely reported as affected. Nevertheless, concerns were expressed regarding veterinary costs, leaving the dog unsupervised, fear of acute adrenal crises, and modifying travel plans to accommodate the dog’s condition. Caregivers were also concerned about potential side effects of medications (25). These findings suggest that QoL may still be affected to some degree, even if not explicitly stated.

Overall, all treatment strategies led to high levels of caregiver satisfaction, with no significant differences between groups. The findings of this study demonstrate that all four treatment approaches (ZP, PP, FP, F) led to comparable improvement in clinical signs.

GI signs are common in dogs with HA. Previous studies have reported a frequency of anorexia in 89%, vomiting in 72%, weight loss in 42%, and diarrhea in 35% of dogs prior to the initiation of treatment (8, 9). In our analysis, GI signs remained relevant during HA treatment, affecting 23–44% of dogs, with no significant differences between treatment groups. These findings can suggest that either persistent hypocortisolism is present, or that these dogs suffered from comorbid conditions. Possible causes for persistent GI signs may include chronic enteropathies or alterations in the gut microbiome. Glucocorticoids play a crucial role in maintaining intestinal barrier integrity. A lack of glucocorticoids can impair this barrier, leading to mucosal erosions, ulcers, and increased susceptibility to gastrointestinal bleeding (61, 62). The generally normal activity levels and QoL of both dogs and caregivers argue against hypocortisolism as the primary cause of GI signs.

Studies of dogs recovering from parvoviral enteritis, which severely damages the intestinal barrier, suggest a higher prevalence of chronic GI signs in these dogs later on, possibly due to barrier dysfunction or previous antibiotic exposure (63). In humans, early antibiotic exposure has long been associated with an increased risk of asthma, allergies, and respiratory disorders in childhood (64–67). Additionally, alterations in the gut microbiota have been linked to the initiation and progression of various diseases, and a balanced interaction between the commensal microbiota and mucosal immune defenses is considered essential (68). Disruptions in this balance have been associated with recurrent *Clostridium difficile* infections, inflammatory bowel disease (IBD), colorectal cancer, non-alcoholic steatohepatitis, type 2 diabetes, obesity, advanced liver disease (69–71), and autoimmune diseases (72).

To date, no studies have investigated the gut microbiome in dogs with HA. However, prior studies in both humans and dogs have shown that changes in fecal microbiota can lead to GI dysbiosis influenced by disease processes (73). In particular diabetic dogs have shown a significantly increased prevalence of *Clostridium difficile* compared to non-diabetic controls (74). Interestingly, diabetic dogs also appear to experience chronic diarrhea more frequently (75). Interestingly, many dogs in this study (24–79%), depending on the treatment group, had received multiple courses of antibiotics. In human medicine, there is evidence that antibiotic therapy can induce both transient (76) and long-lasting (77–79) alterations in the gut microbiome, suggesting that similar changes may have occurred in these dogs as well. Another consideration is that a compromised intestinal barrier due to HA may also interfere with oral tolerance, potentially promoting the development of food hypersensitivities (80, 81). Since the initiation of HA therapy, caregivers reported an improvement of gastrointestinal signs after dietary modification in nearly 80% of dogs, while 12% remained unchanged and 8.8% showed worsening signs. In a previous study, only about 50% of dogs with chronic enteropathy achieved good control of clinical signs 10 days after a food change (26). The difference between the results of our study and previous reports may therefore have several explanations. First, our investigation did not require complete resolution of signs but considered clinical improvement as an endpoint. Second, in the comparative study, classification as food-responsive enteropathy required clinical improvement within only ten days. Dogs that might have responded after a longer period of dietary adaptation were not identified. The fact that such a high proportion of dogs exhibited a marked improvement following dietary modification suggests that gastrointestinal signs in dogs with HA should not be attributed solely to HA. Rather, they may be due the framework of a food-responsive enteropathy, potentially facilitated by an impaired intestinal barrier and disturbed oral tolerance at disease onset. This observation highlights the clinical relevance of dietary change in dogs with HA that continue to suffer from chronic gastrointestinal signs during therapy.

Only a few dogs have received cobalamin supplementation in the weeks before or since HA treatment. However, this study did not collect data on whether serum cobalamin concentrations were assessed and whether low levels were diagnosed. Cobalamin supplementation is indicated when serum concentrations are suboptimal (i.e., <400 ng/L), as dogs with chronic inflammatory enteropathy may also benefit from supplementation even in the low-normal range (82, 83). Even suboptimal cobalamin levels can impair the treatment of the underlying disease, as cobalamin is essential for numerous cellular functions and for the regeneration of the intestinal mucosa. A deficiency can lead to inflammatory mucosal infiltration and villous atrophy (84, 85).

The clinical signs of PD and PU can be manifestations of HA itself or may occur because of iatrogenic hypercortisolism, or due to excessive mineralocorticoid effects in combination with existing hypernatremia and/or hypokalemia. In this study, PU/PD occurred in a relevant proportion of dogs since and during therapy (33–65%) with no significant effect since the initiation of HA treatment, making the interpretation of this finding challenging and could reflect the effect of HA as the cause prior diagnosis. Since activity, and QoL were generally rated as normal, the presence of PU/PD in dogs during treatment could indicate glucocorticoid excess. This would be particularly surprising given the relatively low median prednisolone doses used in all groups. However, dogs in the PP group received the lowest prednisolone dosages that could explain that these dogs were less frequently affected by PU compared to the other groups. Polyphagia may reflect either normalization of appetite or a side effect of excessive glucocorticoid exposure. Polyphagia occurred roughly in the same relevant proportion as PU/PD, affecting approximately 36–65% of dogs during treatment. As expected, it was not evident before HA treatment in any of the groups. These findings suggest that both PU/PD and polyphagia may be dose-dependent side effects of glucocorticoids, and that even minor deviations within the therapeutic range may influence their expression. Again, there was less polyphagia in the PP group – likely to the lower prednisolone dose in this group. PU/PD and polyphagia may constitute relevant issues in long-term management. This highlights the need for carefully individualized glucocorticoid dosing, tailored to the clinical response of each patient. Hupfeld et al. (25) specifically investigated glucocorticoid-associated side effects, though treatment strategy was not analyzed in detail. In that study, 64% of caregivers observed side effects since starting prednisolone, while 35.5% did not. The most frequently reported side effects were PU/PD (26%), polyphagia (17%), coat changes (11%), excessive panting (9%), and weight gain (8%). Less common effects included dry skin, muscle wasting, and weight loss.

When using fludrocortisone, signs of glucocorticoid excess may develop, even if serum electrolytes remain poorly controlled (18). This makes fludrocortisone therapy more challenging to manage. It has been reported that inadequate mineralocorticoid replacement is more common with fludrocortisone than with DOCP (18, 40). Achieving optimal balance between glucocorticoid and mineralocorticoid therapy is not always possible. Some dogs may develop iatrogenic hypercortisolism when fludrocortisone doses are sufficient to normalize serum electrolytes (18). The most commonly reported side effects of fludrocortisone are PU/PD, polyphagia, and panting, attributed to its glucocorticoid activity (17). Notably, among the three prednisolone-treated groups, the FP group received the highest median dose of 0.08 mg/kg/day. Since DOCP is licensed for veterinary use, other medications with mineralocorticoid activity, such as fludrocortisone tablets, may only be used under legal cascade regulations.

A genetic component is suspected in primary HA, as certain breeds such as Poodles, Portuguese Water Dogs, Bearded Collies, and Nova Scotia Duck Tolling Retrievers appear predisposed to the disease (2, 6, 86–89). However, the mode of inheritance may vary between breeds. In this study, Poodles, Poodle crosses (e.g., Poo’s, Doodles), Retrievers, and Bearded Collies were also overrepresented, consistent with findings from previous publications (90, 91). Of the 244 dogs included, 52% were female (126/244) and 48% were male (118/244). Unlike earlier studies reporting a female predominance (17), our population showed an almost equal distribution of male and female dogs. At the time of questionnaire completion, the median body weight was 23 kg, which is consistent with previous reports and suggests that larger dogs tend to be more frequently affected. Earlier studies suggest that HA typically affects young to middle-aged dogs, with a median age at diagnosis of 3–4 years. In our cohort, the median age at the time of questionnaire completion was 7.0 years, while the median age at diagnosis was 3 years. These data are also consistent with previous reports (13, 44, 92).

In general, the prognosis for dogs with HA is considered very good (43, 46, 93). Both a study from 1997 and a recent study from 2025 demonstrated that the survival time of dogs with HA under adequate therapy does not differ from that of a control cohort (46, 93). Our results even suggest a more favorable outcome compared to previous reports, thereby further supporting the notion that HA does not negatively affect overall life expectancy. In our study, 7% of the dogs were either deceased or had been euthanized. Notably, among those cases, only one dog was reported to have died from HA. Upon closer review, the cause appeared to be caregiver overwhelm due to difficulty managing the disease. Five dogs were reported to have died or been euthanized due to neoplasia. The median age at death among the deceased dogs in this study was 13 years. This findings suggest that HA may not significantly shorten life expectancy in dogs. However, the limited dataset does not allow a definitive assessment of survival time.

This study faces several limitations. Most of the questionnaires were completed by caregivers active in social media groups. Approximately 27 of the 244 dogs were reported to be under the care of a university hospital. As most data were collected from social media groups, the sample does not represent the full population of dogs with HA. Caregivers active in these groups tend to aim for lower glucocorticoid doses.

Concerning the optimal mineralocorticoid dose, some authors recommend using a combination of Plasma-renin-activity (PRA), sodium-to-potassium ratio, assessment of hypertension, and clinical signs to guide DOCP therapy (36). Sieber-Ruckstuhl et al. (19) proposed that electrolytes should remain within the reference range, regardless of the Na: K ratio. In newly diagnosed dogs, PRA levels are significantly higher than in healthy controls (94). In successfully treated dogs, PRA values were comparable to those in healthy dogs and humans (94–96).

While caregivers satisfaction was assessed in this study, more detailed caregiver-related variables such as perceived treatment burden, practical challenges of daily management, emotional stressors, or financial strain were not collected. Previous work has shown that such factors may influence treatment adherence and caregiver well-being, even when the dog’s clinical condition is well controlled (25). Future studies may therefore benefit from including these additional caregiver-centered variables to gain a more comprehensive understanding of the long-term impact of managing primary HA, and their potential influence should be further investigated.

Since members of social media groups are often encouraged to monitor electrolytes regularly, it is likely that sodium and potassium concentrations were within normal limits for most dogs in this study. However, electrolyte values, potential PRA measurements, or blood pressure readings were not collected in this study, and only caregiver-reported clinical signs could be used to evaluate dosing effectiveness. Another limitation was the unequal size of the four treatment groups. This disparity is likely due to availability issues: Percorten-V® is currently only available outside of Europe and was temporarily difficult to obtain in the US. Zycortal® is currently the only globally available licensed product, which explains the large size of the ZP group. Fludrocortisone, a human-labeled product, requires off-label use (cascade regulation) in Europe. It was commonly used before DOCP became widely available, which explains the small size of the F group.

In conclusion, this study demonstrates that all four treatment strategies (ZP, PP, FP, F) for managing primary HA with electrolyte imbalances in dogs are effective in achieving clinical disease control. No significant differences were identified between groups in terms of overall clinical improvement. The DOCP dose used in this study were consistently lower than previously published recommendations, yet this did not impair clinical control. To our knowledge, this is the first study to systematically describe prednisolone in combination with DOCP and similar to prednisone, low prednisolone doses can be combined safely with DOCP. These findings support the potential need to re-evaluate current dosing recommendations, with consideration for lower individualized dosages. Despite successful replacement therapy, approximately one-third of dogs continued to show gastrointestinal signs, with a substantial proportion of affected dogs showing improvement after dietary modification, suggesting that food-responsive enteropathies may play a role in this context. This warrants further investigation in future studies.

## Data Availability

The raw data supporting the conclusions of this article will be made available by the authors, without undue reservation.

## References

[1] Kelch WJ Canine Hypoadrenocorticism (Canine Addison's Disease): History, Contemporary Diagnosis by Practicing Veterinarians, and Epidemiology: The University of Tennessee 1996

[2] Hanson J Tengvall K Bonnett B Hedhammar Å. Naturally occurring adrenocortical insufficiency–an epidemiological study based on a swedish-insured dog population of 525,028 dogs. J Vet Intern Med. (2016) 30:76–84. doi: 10.1111/jvim.13815, 26683136 PMC4913634

[3] Frank CB Valentin SY Scott-Moncrieff JC Miller MA. Correlation of inflammation with adrenocortical atrophy in canine adrenalitis. J Comp Pathol. (2013) 149:268–79. doi: 10.1016/j.jcpa.2012.11.242, 23348017

[4] Peterson ME Kintzer PP Kass PH. Pretreatment clinical and laboratory findings in dogs with hypoadrenocorticism: 225 cases (1979-1993). J Am Vet Med Assoc. (1996) 208:85–91. doi: 10.2460/javma.1996.208.01.85, 8682712

[5] Baumstark ME Sieber-Ruckstuhl NS Muller C Wenger M Boretti FS Reusch CE. Evaluation of aldosterone concentrations in dogs with hypoadrenocorticism. J Vet Internal Med. (2014) 28:154–9. doi: 10.1111/jvim.12243, 24428320 PMC4895548

[6] Oberbauer AM Benemann KS Belanger JM Wagner DR Ward JH Famula TR. Inheritance of hypoadrenocorticism in bearded collies. Am J Vet Res. (2002) 63:643–7. doi: 10.2460/ajvr.2002.63.643, 12013462

[7] Hughes AM Nelson RW Famula TR Bannasch DL. Clinical features and heritability of hypoadrenocorticism in Nova Scotia duck tolling retrievers: 25 cases (1994-2006). J Am Vet Med Assoc. (2007) 231:407–12. doi: 10.2460/javma.231.3.407, 17669043

[8] Scott-Moncrieff JC. Hypoadreocorticism in dogs In: Feldman EC Nelson RW Reusch CE Scott-Moncrieff JC, editors. Canine and Feline Endocrinology, 3rd ed. St. Louis, MO: Elsevier (2014) 485–520.

[9] Scott-Moncrieff JC. Hypoadrenocorticism in dogs. In: Ettinger SJ Feldman EC Côté E editors. Textbook of Veterinary Internal Medicine. 8th ed. St. Louis, MO: Elsevier (2017) 1795–1810.

[10] Adler JA Drobatz KJ Hess RS. Abnormalities of serum electrolyte concentrations in dogs with hypoadrenocorticism. J Vet Intern Med. Elsevier Health sciences (2007) 21:1168–1173. 18196721 10.1892/06-270.1

[11] Gow AG Gow DJ Bell R Simpson JW Chandler ML Evans H . Calcium metabolism in eight dogs with hypoadrenocorticism. J Small Anim Pract. (2009) 50:426–30. 19689671 10.1111/j.1748-5827.2009.00757.x

[12] Hauck C Schmitz SS Burgener IA Wehner A Neiger R Kohn B . Prevalence and characterization of hypoadrenocorticism in dogs with signs of chronic gastrointestinal disease: a multicenter study. J Vet Intern Med. (2020) 34:1399–405. doi: 10.1111/jvim.15752, 32573832 PMC7379021

[13] Kintzer PP Peterson ME. Primary and secondary canine hypoadrenocorticism. Vet Clin North Am Small Anim Pract. (1997) 27:349–57. doi: 10.1016/S0195-5616(97)50036-2, 9076912

[14] Kintzer PP Peterson ME. Diagnosis and management of primary spontaneous hypoadrenocorticism (Addison's disease) in dogs. Semin Vet Med Surg Small Anim. (1994) 9:148–52. 7938935

[15] Bates JA Shott S Schall WD. Lower initial dose desoxycorticosterone pivalate for treatment of canine primary hypoadrenocorticism. Aust Vet J. (2013) 91:77–82. doi: 10.1111/avj.12019, 23438457

[16] Carr AP. How best to treat Addison's disease in dogs? Vet Rec. (2016) 179:96–7. doi: 10.1136/vr.i4052, 27450847

[17] Kintzer PP Peterson ME. Treatment and long-term follow-up of 205 dogs with hypoadrenocorticism. J Vet Internal Med. (1997) 11:43–9. doi: 10.1111/j.1939-1676.1997.tb00072.x, 9127289

[18] Lynn RC Feldman EC Nelson RW. Efficacy of microcrystalline desoxycorticosterone pivalate for treatment of hypoadrenocorticism in dogs. DOCP clinical study group. J Am Vet Med Assoc. (1993) 202:392–6. 8440628

[19] Sieber-Ruckstuhl NS Reusch CE Hofer-Inteeworn N Kuemmerle-Fraune C Muller C Hofmann-Lehmann R . Evaluation of a low-dose desoxycorticosterone pivalate treatment protocol for long-term management of dogs with primary hypoadrenocorticism. J Vet Intern Med. (2019) 33:1266–71. doi: 10.1111/jvim.15475, 30865322 PMC6524388

[20] Novartis Animal Health US, Inc. Package insert for Percorten®-V (desoxycorticosterone pivalate). Greensboro, NC: Novartis Animal Health. (1998).

[21] Dechra Veterinary Products Ltd. Package insert for Zycortal® (desoxycorticosterone pivalate). Overland Park, KS: Dechra. (2016).

[22] McCabe MD Feldman EC Lynn RC Kass PH. Subcutaneous administration of desoxycorticosterone pivalate for the treatment of canine hypoadrenocorticism. J Am Anim Hosp Assoc. (1995) 31:151–5. doi: 10.5326/15473317-31-2-151, 7773761

[23] Schofield I O'Neill DG Brodbelt DC Church DB Geddes RF Niessen SJM. Development and evaluation of a health-related quality-of-life tool for dogs with Cushing's syndrome. J Vet Internal Med. (2019) 33:2595–604. doi: 10.1111/jvim.15639, 31660657 PMC6872869

[24] Niessen SJ Powney S Guitian J Niessen AP Pion PD Shaw JA . Evaluation of a quality-of-life tool for dogs with diabetes mellitus. J Vet Intern Med. (2012) 26:953–61. doi: 10.1111/j.1939-1676.2012.00947.x, 22646241

[25] Hupfeld J Dölle M Volk H Rieder J. Effect of long-term management of hypoadrenocorticism on the quality of life of affected dogs and their owners. Vet Rec. (2022) 191:e1977. doi: 10.1002/vetr.1977, 35941756

[26] Allenspach K Wieland B Grone A Gaschen F. Chronic enteropathies in dogs: evaluation of risk factors for negative outcome. J Vet Intern Med. (2007) 21:700–8. doi: 10.1892/0891-6640(2007)21[700:ceideo]2.0.co;2, 17708389

[27] Bellumori TP Famula TR Bannasch DL Belanger JM Oberbauer AM. Prevalence of inherited disorders among mixed-breed and purebred dogs: 27,254 cases (1995-2010). J Am Vet Med Assoc. (2013) 242:1549–55. doi: 10.2460/javma.242.11.1549, 23683021

[28] Klein SC Peterson ME. Canine hypoadrenocorticism: part I. Can Vet J. (2010) 51:63–9. 20357943 PMC2797351

[29] Novartis Animal Health US. Inc. Freedom of Information Summary for Percorten®-V (desoxycorticosterone pivalate). Rockville, MD: U.S. Food and Drug Administration. (1988) 141–029.

[30] Dechra Veterinary Products Ltd. Freedom of Information Summary for Zycortal® (desoxycorticosterone pivalate). Rockville, MD: U.S. Food and Drug Administration. (2016) 141–444.

[31] European Medicines Agency. CVMP assessment report for Zycortal®. EMEA/V/C/003782/0000. London: European Medicines Agency. (2015).

[32] Baumstark ME Nussberger J Boretti FS Baumstark MW Riond B Reusch CE . Use of plasma renin activity to monitor mineralocorticoid treatment in dogs with primary hypoadrenocorticism: desoxycorticosterone versus fludrocortisone. J Vet Intern Med. (2014) 28:1471–8. doi: 10.1111/jvim.12426, 25274440 PMC4895596

[33] Jaffey JA Nurre P Cannon AB DeClue AE. Desoxycorticosterone pivalate duration of action and individualized dosing intervals in dogs with primary hypoadrenocorticism. J Vet Internal Med. (2017) 31:1649–57. doi: 10.1111/jvim.14828, 28892205 PMC5697171

[34] Pascoe K. Treatment of hypoadrenocorticism in dogs. J Am Vet Med Assoc. (1993) 202:1192–3. 8496071

[35] Münch L Münch M Paul H Miklis A Heinrich M Neiger R. Therapy of primary hypoadrenocorticism in dogs with low dose desoxycorticosterone pivalate. Tierarztliche Praxis Ausgabe K, Kleintiere Heimtiere. (2020) 48:171–5. doi: 10.1055/a-1166-8800, 32557492

[36] Vincent AM Okonkowski LK Brudvig JM Refsal KR Berghoff N Olivier NB . Low-dose desoxycorticosterone pivalate treatment of hypoadrenocorticism in dogs: a randomized controlled clinical trial. J Vet Intern Med. (2021) 35:1720–8. doi: 10.1111/jvim.16195, 34114259 PMC8295656

[37] Medinger TL Williams DA Bruyette DS. Severe gastrointestinal tract hemorrhage in three dogs with hypoadrenocorticism. J Am Vet Med Assoc. (1993) 202:1869–72. doi: 10.2460/javma.1993.202.11.1869, 8320158

[38] Mooney C. Addison's disease (hypoadrenocorticism) in dogs. Eur J Comp Anim Pract. (2007) 17:167–172.

[39] Ramsey I. Diagnosis and treatmnent of canine hypoadrenocorticism. In Pract. (2003) 25:18–25. doi: 10.1136/inpract.25.1.18

[40] Melian C Peterson ME. Diagnosis and treatment of naturally occurring hypoadrenocorticism in 42 dogs. J Small Anim Pract. (1996) 37:268–75. doi: 10.1111/j.1748-5827.1996.tb02377.x, 8805097

[41] Spence SF S Roberts E Ramsey I. A comparison of the ACTH concentrations in dogs with stable hypoadrenocorticism being treated with either fludrocortisone or desoxycortone pivalate and prednisolone In: 27th ECVIM-CA congress proceedings (2017). 167.

[42] Ferguson D Hoenig M. Glucocorticoids, mineralocorticoids, and adrenolytic drugs. In: Riviere JE Papich MG editors. Veterinary pharmacology and therapeutics. Hoboken, NJ: John Wiley & Sons (2018) 729–62.

[43] Lathan P Thompson AL. Management of hypoadrenocorticism (Addison's disease) in dogs. Vet Med. (2018) 9:1–10. doi: 10.2147/VMRR.S146774 PMC6055912 30050862

[44] Feldman E Nelson R. Hypoadrenocorticism (Addison's disease) In: Feldman EC, editor. Canine and feline endocrinology and reproduction (2004)

[45] Feldman EC Peterson ME. Hypoadrenocorticism. Vet Clin North Am Small Anim Pract. (1984) 14:751–66. doi: 10.1016/S0195-5616(84)50079-5, 6091311

[46] Kintzer PP. Hypoadrenocorticism (Addison’s disease) In: Tilley LP Sf, editors. The 5 minute veterinary consult: Williams & Wilkins (1997). 716–7.

[47] Reusch C. Hypoadrenocorticism In: Ettinger SJ Feldmen EC, editors. Textbook of veterinary internal medicine. 5th ed. Philadelphia, PA: Saunders (2000). 1488–99.

[48] Church DB. Canine hypoadrenocorticism. In: Mooney CT Peterson ME editors. BSAVA manual of canine and feline endocrinology. *4th* ed. Gloucester: BSAVA (2012) 156–66.

[49] Lathan P. Hypoadrenocorticism in dogs In: J R, editor. Clinical endocrinology of companion animals. 1st ed. Ames, IA: Wiley-Blackwell (2013) 1–21.

[50] Klein SC Peterson ME. Canine hypoadrenocorticism: part II. Can Vet J. (2010) 51:179–84. 20436864 PMC2808283

[51] Zeugswetter FK Haninger T. Prednisolone dosages in Addisonian dogs after integration of ACTH measurement into treatment surveillance. Tierarztliche Praxis Ausgabe K, Kleintiere Heimtiere. (2018) 46:90–6. doi: 10.15654/TPK-170668 29727896

[52] Favier RP Poldervaart JH van den Ingh TS Penning LC Rothuizen J. A retrospective study of oral prednisolone treatment in canine chronic hepatitis. Vet Q. (2013) 33:113–20. doi: 10.1080/01652176.2013.826881 23937599

[53] Ohta H Morita T Yokoyama N Osuga T Sasaki N Morishita K . Serial measurement of pancreatic lipase immunoreactivity concentration in dogs with immune-mediated disease treated with prednisolone. J Small Anim Pract. (2017) 58:342–7. doi: 10.1111/jsap.12652, 28247954

[54] Sebbag L Mochel JP. Pharmacokinetics of Oral prednisone at various doses in dogs: preliminary findings using a naïve pooled-data approach. Front Vet Sci. (2020) 7:571457. doi: 10.3389/fvets.2020.571457, 33195563 PMC7604266

[55] Seth M Drobatz KJ Church DB Hess RS. White blood cell count and the sodium to potassium ratio to screen for hypoadrenocorticism in dogs. J Vet Internal Med. (2011) 25:1351–6. doi: 10.1111/j.1939-1676.2011.00830.x, 22092627

[56] Thompson AL Scott-Moncrieff JC Anderson JD. Comparison of classic hypoadrenocorticism with glucocorticoid-deficient hypoadrenocorticism in dogs: 46 cases (1985-2005). J Am Vet Med Assoc. (2007) 230:1190–4. doi: 10.2460/javma.230.8.1190, 17501661

[57] Fittschen C Bellamy JE. Prednisone treatment alters the serum amylase and lipase activities in normal dogs without causing pancreatitis. Can J Comp Med. (1984) 48:136–40. 6202383 PMC1236025

[58] Edney AT. Reasons for the euthanasia of dogs and cats. Vet Rec. (1998) 143:114. doi: 10.1136/vr.143.4.114, 9725180

[59] Wojciechowska JI Hewson CJ. Quality-of-life assessment in pet dogs. J Am Vet Med Assoc. (2005) 226:722–8. doi: 10.2460/javma.2005.226.722, 15776944

[60] British Veterinary Association (BVA). Animal Welfare Strategy. (2006). Available online at: https://www.bva.co.uk/uploadedFiles/Content/News,_campaigns_and_policies/Policies/Ethics_and_welfare/BVA-animal-welfare-strategy-feb-2016.pdf (Accessed January 20, 2019).

[61] Filaretova L Podvigina T Bagaeva T Bobryshev P Takeuchi K. Gastroprotective role of glucocorticoid hormones. J Pharmacol Sci. (2007) 104:195–201. doi: 10.1254/jphs.CP0070034, 17598953

[62] McNeill JR Stark RD Greenway CV. Intestinal vasoconstriction after hemorrhage: roles of vasopressin and angiotensin. Am J Phys. (1970) 219:1342–7. doi: 10.1152/ajplegacy.1970.219.5.1342, 4319646

[63] Kilian E Suchodolski JS Hartmann K Mueller RS Wess G Unterer S. Long-term effects of canine parvovirus infection in dogs. PLoS One. (2018) 13:e0192198. doi: 10.1371/journal.pone.0192198, 29547647 PMC5856261

[64] Metsälä J Lundqvist A Virta LJ Kaila M Gissler M Virtanen SM. Prenatal and post-natal exposure to antibiotics and risk of asthma in childhood. Clin Exp Allergy. (2015) 45:137–45. doi: 10.1111/cea.12356 24943808

[65] Ungaro R Bernstein CN Gearry R Hviid A Kolho K-L Kronman MP . Antibiotics associated with increased risk of new-onset Crohn’s disease but not ulcerative colitis: a meta-analysis. Am J Gastroenterol. (2014) 109:1728–38. doi: 10.1038/ajg.2014.246 25223575

[66] Saari A Virta LJ Sankilampi U Dunkel L Saxen H. Antibiotic exposure in infancy and risk of being overweight in the first 24 months of life. Pediatrics. (2015) 135:617–26. doi: 10.1542/peds.2014-3407, 25825533

[67] Foliaki S Pearce N Björkstén B Mallol J Montefort S von Mutius E. Antibiotic use in infancy and symptoms of asthma, rhinoconjunctivitis, and eczema in children 6 and 7 years old: international study of asthma and allergies in childhood phase III. J Allergy Clin Immunol. (2009) 124:982–9. doi: 10.1016/j.jaci.2009.08.017, 19895986

[68] Becattini S Taur Y Pamer EG. Antibiotic-induced changes in the intestinal microbiota and disease. Trends Mol Med. (2016) 22:458–78. doi: 10.1016/j.molmed.2016.04.003, 27178527 PMC4885777

[69] Wirbel J Pyl PT Kartal E Zych K Kashani A Milanese A . Meta-analysis of fecal metagenomes reveals global microbial signatures that are specific for colorectal cancer. Nat Med. (2019) 25:679–89. doi: 10.1038/s41591-019-0406-6, 30936547 PMC7984229

[70] Duvallet C Gibbons SM Gurry T Irizarry RA Alm EJ. Meta-analysis of gut microbiome studies identifies disease-specific and shared responses. Nat Commun. (2017) 8:1784. doi: 10.1038/s41467-017-01973-8, 29209090 PMC5716994

[71] Lange K Buerger M Stallmach A Bruns T. Effects of antibiotics on gut microbiota. Dig Dis. (2016) 34:260–8. doi: 10.1159/000443360, 27028893

[72] Xu H Liu M Cao J Li X Fan D Xia Y . The dynamic interplay between the gut microbiota and autoimmune diseases. J Immunol Res. (2019) 2019:7546047. doi: 10.1155/2019/7546047, 31772949 PMC6854958

[73] Grześkowiak Ł Endo A Beasley S Salminen S. Microbiota and probiotics in canine and feline welfare. Anaerobe. (2015) 34:14–23. doi: 10.1016/j.anaerobe.2015.04.002, 25863311 PMC7111060

[74] Kwong TC Chau ECT Mak MCH Choy CT Chan LT Pang CK . Characterization of the gut microbiome in healthy dogs and dogs with diabetes mellitus. Animals. (2023) 13. doi: 10.3390/ani13152479, 37570288 PMC10417117

[75] Frías Ordoñez JS Otero Regino W. Chronic diarrhea in the diabetic. A review of the literature. Rev Gastroenterol Peru. (2016) 36:340–9. 28062871

[76] Palleja A Mikkelsen KH Forslund SK Kashani A Allin KH Nielsen T . Recovery of gut microbiota of healthy adults following antibiotic exposure. Nat Microbiol. (2018) 3:1255–65. doi: 10.1038/s41564-018-0257-9, 30349083

[77] Dethlefsen L Relman DA. Incomplete recovery and individualized responses of the human distal gut microbiota to repeated antibiotic perturbation. Proc Natl Acad Sci USA. (2011) 108:4554–61. doi: 10.1073/pnas.1000087107, 20847294 PMC3063582

[78] Jernberg C Löfmark S Edlund C Jansson JK. Long-term impacts of antibiotic exposure on the human intestinal microbiota. Microbiology. (2010) 156:3216–23. doi: 10.1099/mic.0.040618-0, 20705661

[79] Isaac S Scher JU Djukovic A Jiménez N Littman DR Abramson SB . Short- and long-term effects of oral vancomycin on the human intestinal microbiota. J Antimicrob Chemother. (2017) 72:128–36. doi: 10.1093/jac/dkw383, 27707993 PMC5161046

[80] Chehade M Mayer L. Oral tolerance and its relation to food hypersensitivities. J Allergy Clin Immunol. (2005) 115:3–12. doi: 10.1016/j.jaci.2004.11.008, 15637539

[81] Pabst O Mowat AM. Oral tolerance to food protein. Mucosal Immunol. (2012) 5:232–9. doi: 10.1038/mi.2012.4, 22318493 PMC3328017

[82] Berghoff N Suchodolski JS Steiner JM. Association between serum cobalamin and methylmalonic acid concentrations in dogs. Vet J. (2012) 191:306–11. doi: 10.1016/j.tvjl.2011.03.005 21478039

[83] Toresson L Steiner JM Spodsberg E Olmedal G Suchodolski JS Lidbury JA . Effects of oral versus parenteral cobalamin supplementation on methylmalonic acid and homocysteine concentrations in dogs with chronic enteropathies and low cobalamin concentrations. Vet J. (2019) 243:8–14. doi: 10.1016/j.tvjl.2018.11.004, 30606444

[84] Weiss DJ Wardrop KJ. Schalm's veterinary hematology John Wiley & Sons (2011).

[85] Berghoff N Steiner JM. Laboratory tests for the diagnosis and management of chronic canine and feline enteropathies. Vet Clin North Am Small Anim Pract. (2011) 41:311–28. doi: 10.1016/j.cvsm.2011.01.001, 21486638

[86] Oberbauer AM Bell JS Belanger JM Famula TR. Genetic evaluation of Addison's disease in the Portuguese water dog. BMC Vet Res. (2006) 2:15. doi: 10.1186/1746-6148-2-15, 16670022 PMC1481556

[87] Famula TR Belanger JM Oberbauer AM. Heritability and complex segregation analysis of hypoadrenocorticism in the standard poodle. J Small Anim Pract. (2003) 44:8–12. doi: 10.1111/j.1748-5827.2003.tb00096.x, 12570345

[88] Hughes AM Bannasch DL Kellett K Oberbauer AM. Examination of candidate genes for hypoadrenocorticism in Nova Scotia duck tolling retrievers. Vet J. (2011) 187:212–6. doi: 10.1016/j.tvjl.2009.10.012, 19931476

[89] Decome M Blais MC. Prevalence and clinical features of hypoadrenocorticism in great Pyrenees dogs in a referred population: 11 cases. Can Vet J. (2017) 58:1093–9. 28966360 PMC5603917

[90] Friedenberg SG Lunn KF Meurs KM. Evaluation of the genetic basis of primary hypoadrenocorticism in standard poodles using SNP array genotyping and whole-genome sequencing. Mammalian Genome. (2017) 28:56–65. doi: 10.1007/s00335-016-9671-6, 27864587 PMC5495560

[91] Gershony LC Belanger JM Hytönen MK Lohi H Famula TR Oberbauer AM. Genetic characterization of Addison’s disease in bearded collies. BMC Genomics. (2020) 21:833. doi: 10.1186/s12864-020-07243-0, 33243158 PMC7690126

[92] Herrtage M. Hypoadenocorticism. In: Ettinger SJ Feldman EC editors. Textbook Vet Internal Med. *6th* ed. St. Louis, MO: Elsevier Saunders. (2005) 1595–1606.

[93] Sherrod TN Lashnits E Lunn KF. Clinical characteristics, treatment, and outcomes of hypoadrenocorticism in dogs. J Small Anim Pract. (2025) 66:627–35. doi: 10.1111/jsap.13870, 40237295 PMC12417096

[94] Javadi S Galac S Boer P Robben JH Teske E Kooistra HS. Aldosterone-to-renin and cortisol-to-adrenocorticotropic hormone ratios in healthy dogs and dogs with primary hypoadrenocorticism. J Vet Intern Med. (2006) 20:556–61. doi: 10.1892/0891-6640(2006)20[556:AACHRI]2.0.CO;2 16734089

[95] Oelkers W L'Age M. Control of mineralocorticoid substitution in Addison's disease by plasma renin measurement. Klin Wochenschr. (1976) 54:607–12. doi: 10.1007/BF01469025, 940296

[96] Nussberger J Fasanella d'Amore T Porchet M Waeber B Brunner DB Brunner HR . Repeated administration of the converting enzyme inhibitor cilazapril to normal volunteers. J Cardiovasc Pharmacol. (1987) 9:39–44. 2434792

