# Supplementary material for: Efficacy and side effects of long-term treatment strategies of canine primary hypoadrenocorticism
Source: Front Vet Sci. 2026 Jan 5;12:1735233. doi: 10.3389/fvets.2025.1735233 (PMC12812539; doi:10.3389/fvets.2025.1735233)
Supplement: Supplementary file 1 [file Data_Sheet_1.PDF]

Supplementary Material

1 Supplementary Data

2 Supplementary Figures and Tables

Table S1: Assessment of Disease Control: Activity, Quality of Life (QoL), and Satisfaction

This table presents a detailed analysis of the estimated marginal means for various signs across the different treatment groups (ZP, PP, FP, F). For each sign – reduced activity, and quality of life for both dogs and owners, as well as satisfaction with treatment – the corresponding p-values within each group for presence of absence of a problem are provided. Odds ratios and p-values are also displayed to show comparative significance between treatment groups.

	ZP	PP	FP	F	Comparison between groups<br>Odds-Ratio (OR)<br>p-value[1]

**Reduced activity**	**n[2] = 165**<br><br>estimated marginal means:<br><br>yes: 23/165 (13.94 %)<br><br>no: 142/165 (86.06%)<br><br>p-value: <0.0001*	**n = 39**<br><br>estimated marginal means:<br><br>yes: 1/39 (2.56%)<br><br>no: 38/39 (97.44 %)<br><br>p-value: <0.0001*	**n = 22**<br><br>estimated marginal means:<br><br>yes: 3/22 (13.63%)<br><br>no: 19/22 (86.37%)<br><br>p-value: 0.0018*	**n = 15**<br><br>estimated marginal means:<br><br>yes: 2/15 (13.33%)<br><br>no: 13/15 (86.67%)<br><br>p-value: 0.0024*	*F vs[3] FP:*<br><br>*OR: 0.87*<br><br>*p-value: 0.99*<br><br>*F vs PP:*<br><br>*OR: 3.92*<br><br>*p-value: 0.52*<br><br>*F vs ZP:*<br><br>*OR: 0.840*<br><br>*p-value: 0.99*
**Reduced QoL[4] (dog)**	**n = 167**<br><br>estimated marginal means:<br><br>yes: 18/167 (10.78%)<br><br>no: 149/167 (89.22%)<br><br>p-value: <0.0001*	**n = 39**<br><br>estimated marginal means:<br><br>yes: 1/39 (2.56%)<br><br>no: 38/39 (97.44%)<br><br>p-value: <0.0001*	**n = 23**<br><br>estimated marginal means:<br><br>yes: 6/23 (26.1%)<br><br>no: 17/23 (73.9%)<br><br>p-value: 0.0186*	**n = 15**<br><br>estimated marginal means:<br><br>yes: 1/15 (6.67%)<br><br>no: 14/15 (93.33%)<br><br>p-value: 0.001*	*F vs FP:*<br><br>*OR: 0.26*<br><br>*p-value: 0.39*<br><br>*F vs PP:*<br><br>*OR: 2.56*<br><br>*p-value: 0.8*<br><br>*F vs ZP:*<br><br>*OR: 0.708*<br><br>*p-value: 0.97*

**Reduced QoL (owner)**	**n = 82**<br><br>estimated marginal means:<br><br>yes: 19/82 (23.17%)<br><br>no: 63/82 (76.83%)<br><br>p-value: <0.0001*	**n = 36**<br><br>estimated marginal means:<br><br>yes: 4/36 (11.11%)<br><br>no: 32/36 (88.89%)<br><br>p-value: 0.0002*	**n = 6**<br><br>estimated marginal means:<br><br>yes: 2/6 (33.33%)<br><br>no: 4/6 (66.67%)<br><br>p-value: 0.305	**n = 8**<br><br>estimated marginal means:<br><br>yes: 1/8 (12.5%)<br><br>no: 7/8 (87.5%)<br><br>p-value: 0.028*	*F vs FP:*<br>*OR: 0.47*<br>*p-value: 0.88*<br><br>*F vs PP:*<br>*OR: 0.92*<br>*p-value: 0.99*<br><br>*F vs ZP:*<br>*OR: 0.67*<br>*p-value: 0.95*
**Satisfaction**	**n = 153**<br><br>estimated marginal means:<br><br>yes: 147/153 (96.1%)<br><br>no: 5/153 (3.9%)<br><br>p-value: <0.0001*	**n = 39**<br><br>estimated marginal means:<br><br>yes: 38/39 (97.44%)<br><br>no: 1/39 (2.56%)<br><br>p-value: 0.0001*	**n = 21**<br><br>estimated marginal means:<br><br>yes: 21/21 (100%)<br><br>no: 0/21 (0%)<br><br>p-value: 0.0069*	**n = 14**<br><br>estimated marginal means:<br><br>yes: 14/14 (100%)<br><br>no: 0/14 (0%)<br><br>p-value: 0.0004*	*F vs FP:*<br>*OR: 0.5*<br>*p-value: 0.98*<br><br>*F vs PP:*<br>*OR: 1.68*<br>*p-value: 0.98*<br><br>*F vs ZP:*<br>*OR: 2.37*<br>*p-value: 0.9*

[1]p = level of significance, [2]n = number; [1]p = level of significance;[3]vs = versus; [4]QoL = quality of life

Table S2: Supportive treatment used to treat GI signs

Overview of medications that were applied to treat GI signs in dogs before and since HA specific treatment. The category "other medications" includes analgesics (e.g., metamizole, butylscopolamine, gabapentin), anti-diarrheal agents (e.g., Dia Tab®, charcoal tablets, Perenterol®, Imodium® and "none specified" medications, if no specific substances were documented.

		ZP	PP	FP	F
**Probiotics**	**before**	Yes: 33/84 (39.29%)<br>No: 51/84 (60.71%)	Yes: 8/34 (23.53%)<br>No: 26/34 (76.47%)	Yes: 9/11 (81.82%)<br>No: 2/11 (18.18%)	Yes: 3/7 (42.86%)<br>No: 4/7 (57.14%)
	**since**	Yes 53/95 (55.57%)<br>No: 42/95 (44.43%)	Yes: 10/36 (27.87%)<br>No: 26/36 (72.22%)	Yes: 11/12 (91.67%)<br>No: 1/12 (9.33%)	Yes: 3/7 (42.86%)<br>No: 4/7 (57.14%)
**Antibiotics (e.g. metronidazole, tylosine, amoxicillin)**	**before**	Yes: 33/79 (41.77%)<br>No: 46/79 (58.23%)	Yes: 8/34 (23.53%)<br>No: 26/34 (76.47%)	Yes:11/13 (84.62%)<br>No: 2/13 (15.38%)	Yes: 2/6 (33.33%)<br>No: 4/6 (66.67%)
	**since**	Yes: 41/87 (47.1%)<br>No: 47/87 (52.9%)	Yes: 11/36 (30.56%)<br>No: 25/36 (69.44%)	Yes: 12/14 (85.57%)<br>No: 2/14 (14.43%)	Yes: 4/6 (66.67%)<br>No: 2/6 (33.33%)
**Cobalamin supplementation**	**before**	Yes: 3/70 (4.29%)<br>No: 67/70 (95.71%)	Yes: 0/34<br>No: 34/34 (100%)	Yes: 1/6 (16.7%)<br>No: 5/6 (83.3%)	Yes: 0/6<br>No: 6/6 (100%)
	**since**	Yes: 5/72 (6.9%)	Yes: 1/36 (2.78%)	Yes: 1/6 (16.67%)	Yes 1/7 (14.29%)

		No: 67/72 (93.1%)	No: 35/36 (97.22%)	No: 5/6 (83.33%)	No: 6/7 (85.71%)
**Gastric protection**	**before**	Yes: 0/0<br>No: 0/0	Yes: 0/0<br>No: 0/0	Yes: 0/0<br>No: 0/0	Yes: 0/0<br>No: 0/0
	**since**	Yes: 5/6 (83.3%)<br>No: 1/6 (16.7%)	Yes: 0/0<br>No: 0/0	Yes: 0/0<br>No: 0/0	Yes: 0/0<br>No: 0/0
**Other medication**	**before**	Yes: 14/72 (19.44%)<br>No: 58/72 (80.56%)	Yes: 5/34 (14.71%)<br>No: 29/34 (85.29%)	Yes: 4/8 (50%)<br>No: 4/8 (50%)	Yes: 3/8 (37.50%)<br>No: 5/8 (62.50%)
	**since**	Yes: 31/87 (39.3%)<br>No: 56/87 (60.06%)	Yes: 6/36 (16.67%)<br>No: 30/36 (83.33%)	Yes: 6/17 (35.29%)<br>No: 11/17 (64.71%)	Yes 3/8 (37.50%)<br>No: 5/8 (62.50%)

		ZP	PP	FP	F
**Probiotics**	**before**	Yes: 34/88 (38.6%) No: 54/88 (61.4%)	Yes: 8/34 (23.53%) No: 26/34 (76.47%)	Yes: 9/12 (75%) No: 3/12 (15%)	Yes: 3/7 (42.9%) No: 4/7 (57.1%)
	**since**	Yes 38/68 (55.9%) No: 30/68 (44.1%)	Yes: 5/16 (31.3%) No: 11/16 (68.7%)	Yes: 6/7 (85.7%) No: 1/7 (14.3%)	Yes: 0/2 (0%) No: 2/2 (100 %)
**Antibiotics (e.g. metronidazole, tylosine, amoxicillin)**	**before**	Yes: 34/84 (40.5%) No: 50/84 (59.5%)	Yes: 8/34 (23.5%) No: 26/34 (76.5%)	Yes:11/14 (78.6%) No: 3/14 (21.4%)	Yes: 2/6 (33.33%) No: 4/6 (66.67%)
	**since**	Yes: 21/58 (36.2%) No: 37/58 (63.8%)	Yes: 7/16 (43.8%) No: 9/16 (56.3%)	Yes: 5/7 (71.4%) No: 2/7 (28.6%)	Yes: 2/3 (66.67%) No: 1/3 (33.33%)
**Cobalamin supplementation**	**before**	Yes: 3/74 (4.1%) No: 71/74 (95.9%)	Yes: 0/35 (0%) No: 35/35 (100%)	Yes: 1/6 (16.7%) No: 5/6 (83.3%)	Yes: 0/6 (0%) No: 6/6 (100%)
	**since**	Yes: 4/52 (7.7 %) No: 48/52 (92.3%)	Yes: 1/16 (2.78%) No: 15/16 (97.22%)	Yes: 0/3 (0%) No: 3/3 (100%)	Yes 1/3 (33.33%) No: 2/3 (66.67%)

**Gastric protection**	**before**	Yes: 6/7 (85.7%)<br>No: 1/7 (14.3%)	Yes: 0/0<br>No: 0/0	Yes: 1/1 (100%)<br>No: 0/1	Yes: 0/0<br>No: 0/0
	**since**	Yes: 11/1 (100%)<br>No: 0/11 (0%)	Yes: 0/0<br>No: 0/0	Yes: 1/1 (100%)<br>No: 0/1 (0%)	Yes: 0/0<br>No: 0/0
**Other medication**	**before**	Yes: 20/79 (25.3%)<br>No: 59/79 (74.7%)	Yes: 5/34 (14.7%)<br>No: 29/34 (85.3%)	Yes: 5/9 (55.6%)<br>No: 4/9 (44.4%)	Yes: 3/8 (37.5%)<br>No: 5/8 62.5%)
	**since**	Yes: 28/66 (42.4%)<br>No: 38/66 (57.6%)	Yes: 1/15 (6.7%)<br>No: 14/15 (93.3%)	Yes: 4/6 (66.7%)<br>No: 2/6 (33.3%)	Yes 1/3 (33.33%)<br>No: 2/3 (66.67%)

Table S3: Selected clinical signs since therapy

Frequencies of polydipsia (PD), polyuria (PU), and polyphagia since therapy across the treatment groups ZP, PP, FP, and F. Shown are the number and percentage of dogs affected per group, along with group-specific *p*-values derived from logistic regression models.

	**ZP**	**PP**	**FP**	**F**	**Comparison between groups**<br>**Odds-Ratio (OR)**<br>**p-value**[1 ‡]

**PD**<br><br>**since therapy**	**n[2] = 164**<br><br>estimated marginal means:<br><br>yes: 78/164 (47.56%)<br><br>no: 86/164 (52.4%)<br><br>*p-value: 0.531*	**n = 39**<br><br>estimated marginal means:<br><br>yes: 18/39 (46.15%)<br><br>no: 21/39 (53.85%)<br><br>*p-value: 0.631*	**n = 23**<br><br>estimated marginal means:<br><br>yes: 15/23 (65.22%)<br><br>no: 8/23 (34.78%)<br><br>*p-value:* 0.164	**n = 15**<br><br>estimated marginal means:<br><br>yes: 7/15 (46.67%)<br><br>no: 8/15 (53.33%)<br><br>*p-value: 0.85*	*F vs FP:*<br><br>*OR: 0.5*<br><br>*p-value: 0.68*<br><br>*F vs PP:*<br><br>*OR: 1.07*<br><br>*p-value: 1.00*<br><br>*F vs ZP:*<br><br>*OR: 1.01*<br><br>*p-value: 1.00*
**PU**<br><br>**since therapy**	**n = 159**<br><br>estimated marginal means:<br><br>yes: 71/159 (44.65%)<br><br>no: 88/159 (55.35%)<br><br>p-value: 0.172	**n = 39**<br><br>estimated marginal means:<br><br>yes: 13/39 (33.33%)<br><br>no: 26/39 (66.67 %)<br><br>p-value: 0.034*	**n = 23**<br><br>estimated marginal means:<br><br>yes: 12/23 (52.17%)<br><br>no: 11/23 (47.82%)<br><br>p-value: 0.887	**n = 14**<br><br>estimated marginal means:<br><br>yes: 5/14 (35.71%)<br><br>no: 9/14 (64.39%)<br><br>p-value: 0.287	*F vs FP:*<br><br>*OR: 0.56*<br><br>*p-value: 0.79*<br><br>*F vs PP:*<br><br>*OR: 1.18*<br><br>*p-value: 0.99*<br><br>*F vs ZP:*<br><br>*OR: 0.74*<br><br>*p-value: 0.93*

**polyphagia since therapy**	**n = 166**<br>estimated marginal means:<br>yes: 101/166 (60.84%)<br>no: 65/166 (39.16%)<br>p-value: 0.0056*	**n = 39**<br>estimated marginal means:<br>yes: 14/39 (35.9%)<br>no: 25/39 (64.1%)<br>p-value: 0.089	**n = 23**<br>estimated marginal means:<br>yes: 15/23 (65.22%)<br>no: 8/23 (34.78%)<br>p-value: 0.156	**n = 15**<br>estimated marginal means:<br>yes: 8 /15 (53.33%)<br>no: 7/15 (46.67%)<br>p-value: 0.763	*F vs FP:*<br>*O : 0.63*<br>*p-value: 0.88*<br>*F vs PP:*<br>*OR: 2.01*<br>*p-value: 0.6*<br>*F vs ZP:*<br>*OR: 0.74*<br>*p-value: 0.9*

[1]p = level of significance; [2]n = number
